# Characterization and phylogenetic analysis of the mitochondrial genome of *Schizothorax taliensis*, a national-protected fish in China

**DOI:** 10.1080/23802359.2018.1476071

**Published:** 2018-05-25

**Authors:** Xiao Rui, Xiao-Qing Sun, Shang-Qi Li, Jiong-Tang Li

**Affiliations:** a Fisheries College, Zhejiang Ocean University, Zhoushan, Zhejiang, China;; b Key Laboratory of Aquatic Genomics, Ministry of Agriculture, CAFS Key Laboratory of Aquatic Genomics and Beijing Key Laboratory of Fishery Biotechnology, Chinese Academy of Fishery Sciences, Beijing, China

**Keywords:** *Schizothorax taliensis*, endangered fish, mitochondrial genome

## Abstract

*Schizothorax taliensis* is a national key protected fish in China. The mitochondrial genome of *S. taliensis* is 16,578 bp in length and includes two ribosomal RNA genes, 22 tRNA genes, and 13 protein-coding genes. The phylogenetic analysis showed that *S. taliensis* belongs to *Cyprinidae* family and is closely related to other *Schizothorax* fish. This mitogenome will contribute to the further conservation and genetic studies of this endangered fish.

*Schizothorax taliensis* is narrowly distributed in Erhai lake and Lancang River, Yunnan province (Chen et al. 2016). Due to the threaten from human activity and exotic fish, it is near extinction (Chen 2013) and has been a national key protected species in China (Wang et al. 2009). To provide an efficient tool to study the biodiversity and conservation of this fish, we sequenced its mitogenome.

The fish was collected in Erhai lake (25.7166 N and 100.2210 E), Yunnan province. The genomic DNA was extracted from the fins. The specimen and the corresponding extracted DNA samples were stored at –70 °C in our laboratory (Centre for Applied Aquatic Genomics, Chinese Academy of Fishery Sciences). A total of 13 pairs of primers, designed based on the mitochondrial genome of *S. biddulphi* (GenBank Accession: NC_017873), were used to amplify the complete mitochondrial genome. The PCR products were sequenced using Sanger method and further assembled into a sequence with CAP3 (Huang and Madan 1999).

The mitochondrial genome is 16,578 bp in length (GenBank Accession: MH094667). It exhibits a slight A + T bias of 54.94%. Thirteen protein-coding genes (PCGs), 22 tRNA genes, and two rRNA genes are predicted with MITOS (Bernt et al. 2013). All the start codons for PCGs are ATG except *COI* which initiates with the codon of GTG. Eight PCGs use TAA as the stop codon and three PCGs (*ND3*, *ATP8*, and *ATP6*) use TAG as the stop codon. Two PCGs (*ND2* and *ND4*) use AGG as the stop codon.

We constructed a phylogenetic tree including *S. taliensis* and the other 19 fish. For each species, we extracted the 13 PCGs and concatenated them into one sequence. The 20 sequences were aligned with Clustal Omega (Sievers et al. 2011). Based on the alignment, we constructed the phylogenetic tree using maximum likelihood (ML) method in the MEGA package (Kumar et al. 2016) under the Jones–Taylor–Thornton model with 1000 bootstrap replicates. The tree showed that *S. taliensis* was grouped with other *Schizothorax* fish in *Cyprinidae* family and closely related to common carp and crucian carp (Figure 1).

**Figure 1.**
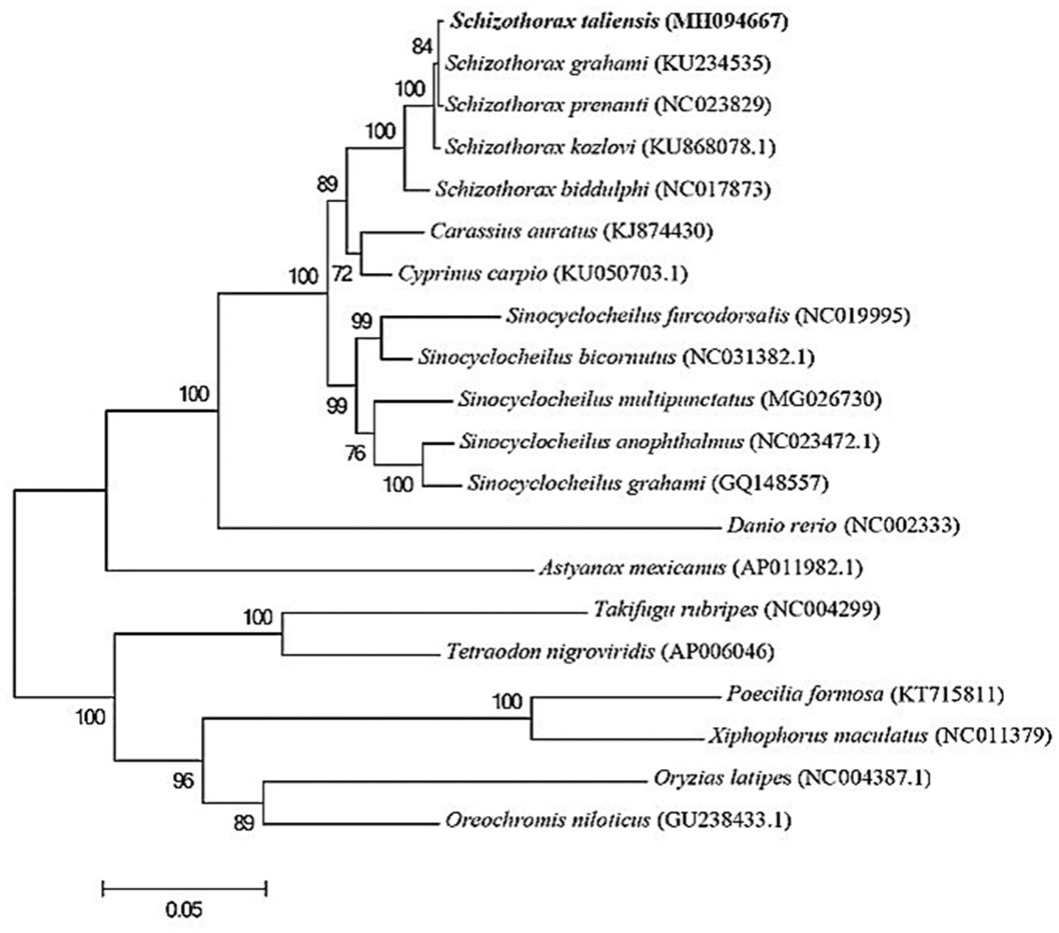
Phylogenetic tree of 20 teleost fish based on 13 mitochondrial protein-coding genes. The number at each node is the bootstrap value of ML analysis.
